# Major Causes of Variation of External Appearance, Chemical Composition, Texture, and Color Traits of 37 Categories of Cheeses

**DOI:** 10.3390/foods11244041

**Published:** 2022-12-14

**Authors:** Giovanni Bittante, Nicolò Amalfitano, Claudio Cipolat-Gotet, Angiolella Lombardi, Giorgia Stocco, Franco Tagliapietra

**Affiliations:** 1 Department of Agronomy, Food, Natural resources, Animals and Environment, University of Padova, 35020 Padua, Italy; 2 Department of Veterinary Science, University of Parma, 43126 Parma, Italy

**Keywords:** cheese survey, cheese technology, cheese types, cheese ripening, goat cheeses

## Abstract

Cheeses are produced by many different procedures, giving rise to many types differing in ripening time, size, shape, chemical composition, color, texture, and sensory properties. As the first step in a large project, our aim was to characterize and quantify the major sources of variation in cheese characteristics by sampling 1050 different cheeses manufactured by over 100 producers and grouped into 37 categories (16 with protected designation of origin, 4 traditional cheese categories, 3 pasta filata cheese categories, 5 flavored cheese categories, 2 goat milk categories, and 7 other categories ranging from very fresh to very hard cheeses). We obtained 17 traits from each cheese (shape, height, diameter, weight, moisture, fat, protein, water soluble nitrogen, ash, pH, 5 color traits, firmness, and adhesiveness). The main groups of cheese categories were characterized and are discussed in terms of the effects of the prevalent area of origin/feeding system, species of lactating females, main cheese-making technologies, and additives used. The results will allow us to proceed with the further steps, which will address the interrelationships among the different traits characterizing cheeses, detailed analyses of the nutrients affecting human health and sensorial fingerprinting.

## 1. Introduction

Cheese is produced throughout the world in thousands of types [1,2] that differ according to the species of lactating females, the cheese-making procedures used (milk pretreatments, the addition and the type of microbial starters, heating, acidification, renneting, curd cutting, cooking, pressing, salting, etc.), the form and size of the cheese, ripening length and conditions, etc. [3]. Almost 20,000 scientific articles with “cheese” in the title have been published in peer-reviewed journals (1 October 2022), and almost all deal with specific aspects of a single or a few types of cheese.

Some surveys have been conducted on many types of nationally produced cheeses dealing with the microbiological and toxicological aspects potentially affecting human health, while others have dealt with fatty acid and mineral profiles [4,5,6], but there is scarce information on those aspects of cheese related to the cheese-making techniques used, the shape and size of cheeses, and their chemical composition and organoleptic properties [7,8]. Stakeholders of the dairy chain frequently express their need for more information and data [9].

There is, in particular, scarce information on the sources of variation in different properties of industrially produced cheeses obtained from a large number of cheese producers and types of cheese. To help answer these questions, a large project, “Caseus Veneti”, was established in northeastern Italy with the aim of characterizing at least 1000 different cheeses produced by more than 100 cheese factories and belonging to at least three dozen different cheese types, with particular reference to those granted Protected Designation of Origin (PDO) status by the European Union [10]. The project is being carried out in five major steps: (i) cheese collection, sampling, analysis, and description; (ii) the development of secondary methods based on the vibrational properties of cheese to predict their chemical and physical properties; (iii) the detailed analysis of nutrients for human health, with special attention to the fatty acid (FA) profile; (iv) quantification of the volatile organic compounds (VOC) in cheese; and (v) a holistic approach to the relationships among all the different features of the cheeses.

This study addresses step one of the project, which serves as the basis for the project’s subsequent steps. Our aims, in particular, are: to report the collection criteria, to list the types, origins, and main cheese-making techniques adopted; to describe the external appearance; to quantify the major sources of variation of the chemical composition, color, and texture of the sampled cheeses; and to discuss these aspects in relation to milk origin and cheese-making procedures.

## 2. Materials and Methods

### 2.1. Experimental Design and Cheese Classification

*Caseus Veneti* is an annual cheese exhibition and competition (https://caseusitaly.it/, accessed on 6 December 2022) sponsored by the Veneto Regional Government (northeastern Italy) and organized by the A.Pro.La.V. (Veneto Dairy Producers Association, https://www.aprolav.com/, accessed on 6 December 2022), which has the aim of assessing and promoting the region’s cheeses and producers. The Veneto dairy sector has a long tradition of producing a wide variety of cheeses. 

Over three annual exhibitions, a total of 1050 different cheeses from 107 producers and grouped into 37 categories (one cheese per producer, per category, and per year) were collected, recorded, sampled, and analyzed.

Due to their wide variation, the cheeses in competition are divided into 37 categories, classified into 3 main groups: PDO cheeses, regional traditional cheeses, and other cheeses. The Veneto region produces 8 PDO cheeses, 7 presented at *Caseus Veneti*, 2 of which are in a single category each (Casatella Trevigiana PDO and Piave PDO cheeses), while the other 5 are divided among two to four categories, mainly according to the length of ripening: 2 categories for Grana Padano PDO and Provolone PDO; 3 categories for Montasio PDO and Monte Veronese PDO; and 4 categories for Asiago PDO. All in all, the 7 PDO cheeses presented were classified in the exhibition into 16 different categories, and 321 individual cheeses were sampled.

The second group comprised four categories recognized by the Veneto Regional Government as “traditional cheeses”: Morlacco del Grappa (produced in the Monte Grappa Massif and Piedmont area), Malga cheeses (produced exclusively during the summer transhumance of cows to high Alpine pastures, recognized if produced in the Belluno province), divided into two categories according to length of ripening (Malga fresco, <12 months and Malga vecchio, >12 months), and the Formaggio Inbriago (literally “drunken cheese”, as it is partly ripened in marc, must, or wine). A total of 136 cheeses in these four categories were sampled.

The cheeses without official recognition of origin (other cheeses) were grouped according to their main technical specifications: 3 “pasta filata” categories (44 cheeses made by stretching the curd at high temperature); 5 “flavored cheese” categories (128 cheeses with spices or herbs added to the milk or curd or on the rind, or smoked, or inoculated with *Penicillium*); 2 “caprino” categories (54 cheeses made from acid- or rennet-coagulated goats’ milk); 7 “other” categories (367 cheeses from very fresh to very hard cheeses). The correlations and independent latent explanatory factors between these categories and relative cheeses are reported in the companion paper of Bittante et al. [11].

### 2.2. Descriptions, Measurements, and Sampling of the Cheeses

The technical features of each cheese presented at the competition were collected by the exhibition organizers and are summarized in Table 1 for PDO cheeses (production of these cheeses is controlled by EU-approved regulations) and in Table 2 for non-PDO cheese categories. Details on the cheese-making procedures of many Italian PDO cheeses presented here can be found in Gobbetti et al. [12].

The norms of production of PDO cheeses are approved by European Union, and, thus, the public information available is more abundant and precise and the variability among cheese producers is much lower than in the case of the other types of cheese.

Upon submission to the exhibition, all individual cheeses were weighed with a technical scale and measured and evaluated for conformity with the relevant PDO regulations or for inclusion in the non-PDO categories. The cheese wheel measures were maximum diameter, circumference, and thickness. In the case of rectangular cuboid cheeses (blocks), the maximum width, height, and length were measured. Each cheese was photographed before and after being cut into two halves.

Immediately after halving, a representative sample of the cheese, either a section of the wheel or a thick central slice of the block of at least 300 g, was taken. In the case of small cheeses (<500 g), the sample consisted of one whole cheese or several cheeses from the same batch. The cheese samples were weighed and immediately vacuum-packed, refrigerated at +4 °C, then transported to the Milk Quality Laboratory of the Department of Agronomy, Food, Natural Resources, Animals and Environment (DAFNAE) of the University of Padova (Legnaro, Italy). Within 3 d of sampling, the packaged samples, beginning with the fresh cheese categories, were opened, and physical measurements were taken; a sample (>50 g) was immediately taken and frozen at −20 °C for chemical analyses.

### 2.3. Cheese Analyses

Cheese analyses were extensively described in a previous study under the same Caseus Veneti project [13]. Briefly, chemical composition (moisture, fat, protein, and ash) was analyzed using the near-infrared (NIR) calibrations for cheese composition preinstalled in a Foodscan spectrometer (FOSS A/S, Hillerød, Denmark). As the Foodscan results for the “Formaggi erborinati” category (blue cheeses) category are unreliable, we used our own calibrations, which were developed on a subset of 197 cheeses selected from the Caseus Veneti collection, representing all the cheese categories and analyzed by gold standard wet analyses. The calibration and validation results are described in detail in our previous articles [13,14]. Our own calibration was also used to determine the content of water-soluble nitrogen (WSN, in % of total nitrogen) in the cheeses.

Color was measured at 3 different positions on the freshly cut surface using a portable spectro-colorimeter (CM 508, Minolta Co., Ltd., Osaka, Japan) standardized with a white calibration cap (CM-A70, Minolta Co., Ltd.). Color traits were expressed according to the Commission Internationale de l’Éclairage colorimetric system (CIE L*a*b*): the L* value is the lightness coefficient (ranging from black = 0 to white = 100); the a* value indicates the position on the green (−) to red (+) axis; the b* value indicates the position on the blue (−) to yellow (+) axis; the C* (chroma) is calculated as $C{\;}^{*}=\sqrt{(a^{2}+b^{2}})$; and the h° (hue angle) values are calculated from the arctangent of b*/a* [15]. The 3 measures from each cheese sample were averaged before statistical analysis. 

Cheese pH was measured in triplicate on all fresh samples using a portable pH meter (Crison Basic 25; Crison Instruments SA, Barcelona, Spain), and the 3 acquisitions were averaged before statistical analysis.

The texture properties of the cheeses were analyzed with a texture analyzer (XT2i, Stable Micro Systems Ltd., Godalming, Surrey, UK) fitted with a Warner–Bratzler shear device (50-N load cell; 2 mm/s crosshead speed). Two cylinder-shaped core samples were taken from each cheese (1 cm^2^ cross-sectional area; 3 cm long), and their textural values were averaged before statistical analysis. Texture data were reported as firmness (defined as the maximum shear force, expressed in Newtons) and adhesiveness (defined as the working shear force, expressed in mJ). 

### 2.4. Statistical Analysis

**Figure 1:**
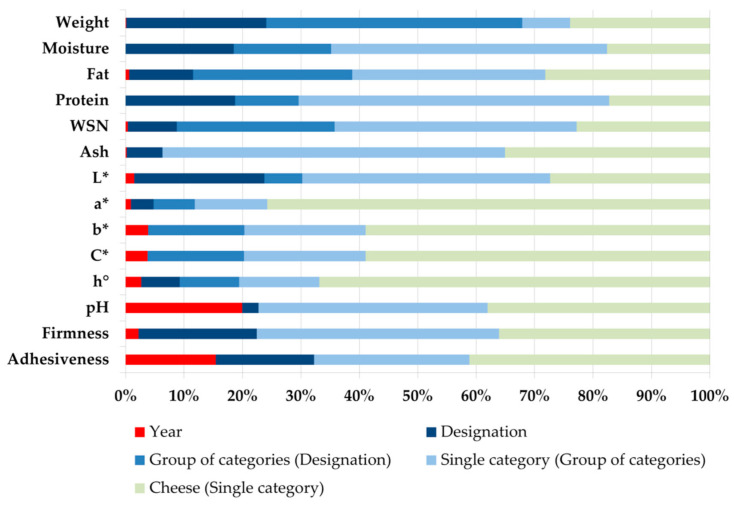
Percentage of total variance of each cheese trait represented by their major sources of variation: the year of sampling, the Designation type (PDO cheeses, Traditional Cheeses, Other cheeses), the Group of categories within Designation (12 groups, see Table 1 and Table 2), the Single categories within groups (37 categories), and the individual cheese within category (1050 cheeses sampled).

As the objective of this study is mainly of a descriptive nature, with the results serving as the basis for further steps in the research project, all the data obtained are reported in the tables as the means ± standard deviations of each trait for every cheese category. This is to allow us to compare the cheese categories not only in terms of their average values but also in terms of the variability among individual cheese categories, without any *a-priori* assumption on their distribution and possible heteroskedasticity among the cheese categories. A linear model including the random effects of year of sampling, designation type (3 levels: PDO, Traditional, or Other cheeses), group of categories within designation (12 groups: 7 PDO groups, 1 traditional cheeses, and 4 other cheeses groups), single cheese category within groups (37 categories) and of individual cheeses within category (residual) was also used for quantifying the major sources of variation of cheese traits. The variance of each of the five random effects was expressed as a percentage of their sum and represented in Figure 1. Specific statistical models will be used in the subsequent steps of the project, according to their objectives.

## 3. Results

### 3.1. Shape and Size of the Cheeses

The first group of characteristics considered were the shapes and sizes of the cheeses sampled. These are shown in Table 3 for cheeses with EU Protected Designation of Origin (PDO) status (measurements are averages plus standard deviations).

Almost all of the PDO cheeses (14 out of 16 categories) had the classical cylindrical shape (wheels); only 2 categories had a pear shape: the two categories of Provolone Valpadana PDO (short- and medium-matured). The very fresh Casatella Trevigiana PDO were small, the two categories of Grana Padano PDO hard cheese were very large, and the other 5 PDO cheeses (13 categories) were intermediate in size.

The shapes and sizes of the non-PDO cheese categories are summarized in Table 4.

Here, too, the large majority of cheeses were cylindrical in shape (wheels), with the exception of some categories that had different shapes according to different producers. This is especially the case for cheeses that are too soft to keep a defined shape (*freschi* and *freschissimi* cheeses), those that are very small (acid-coagulated goat cheeses and Mozzarella), and other pasta filata and traditional inbriago cheeses. Non-PDO cheeses are generally much smaller than PDO cheeses and are very varied in size, both among and within categories. From Figure 1 it is possible to see that the Group of categories within Designation type is the major source of variation (over 40% of total variance) in cheese weight. In fact, within the single designation it is possible to find groups of categories with very different weight, as in the case of the PDO cheeses where there are very heavy cheeses such as the Grana Padano (over 30 kg) and very light cheeses such as the Casatella Trevigiana (around 1 kg). The Designation and the individual cheese represent a similar proportion (20–25%), while the single category within group represents less than 10%.

### 3.2. Chemical Composition of Cheeses

The chemical compositions of PDO cheeses are summarized in Table 5. The values are in agreement with those established by the production regulations [12]. Moisture was high only in the case of Casatella Trevigiana PDO very fresh rindless cheese and much lower for all the other PDO cheeses, particularly those long-ripened and of average size (Asiago stagionato stravecchio, Montasio stagionato, Monte Veronese d’allevo vecchio, and Piave). As expected, all the other chemical traits (fat, protein, WSN, and ash contents) exhibited the reverse pattern to moisture.

The chemical compositions of the non-PDO cheeses are summarized in Table 6.

Here, too, there was very large variability in the chemical components of the cheeses, both among and especially within cheese categories (Figure 1). It is worth noting that the cheese category with the highest moisture content (and lowest of all the other chemical components representing cheese dry matter) was the acid-coagulated goat cheese. The other non-PDO cheese categories with water accounting for, on average, more than half their weight were the traditional Morlacco del Grappa cheese, the fresh pasta filata Mozzarella cheese, and the category composed of small, fresh, and very fresh (freschi, freschissimi) cheeses. Again, the other chemical components generally followed the reverse pattern to that of moisture.

### 3.3. Cheese Color

The color traits of the paste of the PDO cheeses are summarized in Table 7.

The very fresh Casatella Trevigiana PDO cheese had a very high lightness value (almost white), which decreased with cheeses having longer ripening, regardless of the size of the wheels. The a* color index was always very low and had a central tendency in all categories, except Monte Veronese latte intero cheese (almost null), which had negative values, i.e., tending very slightly to green rather than to red. The differences among the cheese categories for this index were, however, very modest. In contrast, the b* color index was much higher and more positive (yellowness) and more variable in all the PDO cheese categories. The semi-soft PDO cheeses (Asiago fresco, Montasio fresco, Monte Veronese latte intero, and Provolone dolce) had greater yellowness, and Piave PDO cheese lower yellowness. Due to the very low a* index for all cheeses, chroma (C*) was very similar to the b* index for all categories. Hue angle (h°) was generally slightly larger than 90°, and only Piave PDO cheese reached 100°, being the cheese with the highest negative a* and the lowest positive b* indices. It is worth noting that for color traits, except L*, the major source of variation is represented by individual cheese within category (Figure 1).

The color traits of non-PDO cheese categories are summarized in Table 8.

Non-PDO cheeses generally had higher lightness values than PDO cheeses, the highest L* values being for Mozzarella, acid-coagulated goat cheese, and fresh and very fresh cheeses, i.e., those with the highest moisture contents. The a* index was also very low for these cheese categories and was almost always negative, and it exhibited low variability among and within categories, whereas b* was higher and positive (yellowness), and there were much larger differences among categories. The two goat cheese categories and Mozzarella had the lowest b* (and C*) index, i.e., the whiter cheeses. The two goat cheese categories were also those with the highest h° (100°), while the other non-PDO categories had h° indices between 89° and 98°.

### 3.4. Acidity and Texture of Cheeses

The acidity and textural traits of PDO cheeses are summarized in Table 9.

Multi-categories of every PDO cheese exhibited a pattern of increased pH with increasing length of ripening. The two textural traits (firmness and adhesiveness) followed the same pattern, the soft and very soft cheeses (Asiago fresco, Casatella Trevigiana, and Monte Veronese latte intero) having the lowest values.

The same traits for non-PDO cheeses are summarized in Table 10.

In the case of pH, two categories exhibited extreme mean values compared with the other 35 cheese categories: the acid-coagulated goat cheeses (*Caprino coagulazione acida*) had, as expected, the lowest average pH, and the blue cheeses (*Formaggi erborinati*) the highest. The two textural traits were also affected by large variability among and within cheese categories, with a tendency, as expected, to increase with increasing length of ripening and decreasing moisture content.

## 4. Discussion

### 4.1. Variability in Size and Compositional and Physical Traits of Cheeses

The analysis of the major sources of variation of cheese characteristics, summarized in Figure 1, showed clearly that different traits are affected differently. The great subdivision among PDO cheeses, traditional cheeses, and other cheeses differentiates largely the weight of cheeses, their textural traits) and the lightness of cheese paste. This reflects mainly the difference between the majority of the PDO cheeses [16], which are often ripened for a long time with respect to other cheeses, more often fresh or even very fresh. The group of categories within designation type, on the contrary, affects more heavily the weight of cheese, moderately the chemical composition (except ash content) and color traits and in a negligible way, the textural traits, the ash content [7], and the pH of cheese paste. The variability among the categories belonging to the same group affects moderately weight, color (except lightness), pH, and textural traits and highly the composition traits, while the variability among different cheeses in the same category affects the same traits in complementary way (Figure 1).

It is well known that especially PDO cheeses should conform to strict norms regarding their external appearance (and particularly size and weight) and not only the origin and characteristics of milk and the processing technology [10]. As it can be seen from Table 1, a PDO cheese represents a group of categories diversified by their ripening length. In this case, the group of categories is characterized by similar size and weight, but different categories of that PDO cheese present different textural, chemical, and color characteristics. In any case, with the only exception of weight, the differences among different cheese categories within each group of categories are the most important source of variation of cheese characteristics and, particularly, in the case of ash content and paste pH.

The primary driver of variability between and within cheese categories is, as expected, length of ripening [17]. This is obviously due, in part, to the direct effects of ripening: paste drying, fermentation, enzymatic activity, increases in lipolysis and proteolysis products and in soluble nitrogenous compounds, etc. [18]. However, the main differences among cheeses of different ripening lengths are due to the cheese-making techniques adopted [19]. Generally, cheeses to be ripened over a long period are larger (for slower drying), are made with partly skimmed milk, undergo a curd-cooking phase, and have a higher ash content [20]. So, when examining the differences among different types of cheeses, the first thing to consider should be the effect of length of ripening [21].

### 4.2. Characteristics of Mountain Cheeses

Mountain cheeses generally have the advantage of favorable consumer response. Four of the seven PDO cheeses and three of the four traditional cheeses have a “mountain” name and were developed in mountainous areas. Several other non-PDO cheeses in different cheese categories are also produced in mountainous areas, but do not benefit from a geographical indication. The fact that the name and the origin of the cheese are associated with mountains, does not mean they are necessarily produced in the mountains. For example, among the PDO cheeses, the majority of Asiago and Montasio cheeses (names from mountain areas) are produced in the plains of the Piedmont region of the eastern Italian Alps. In contrast, Monte Veronese and Piave PDO cheeses and the Morlacco traditional cheese are produced mainly in hilly and mountainous areas. Of course, mountain milk can be produced under a variety of farming systems, from the most traditional (tied cows fed mainly on hay and some concentrates, and at pasture in summer) to modern intensive ones (indoor farming using total mixed rations with a high level of concentrates), similar to many farms in the plains. These different farming systems affect the nutritional values and sensory properties of mountain cheeses.

In the case of traditional cheeses, Malga fresco and Malga vecchio are produced exclusively from milk from cows grazed on Alpine highland pasture (*alpeggio* in Italian, *alm* in German, *alpage* in French) during the summer transhumance from lowland permanent farms to temporary Alpine farms (known as *malga* in Italian, *alm hütte* in German, *chalet d’alpage* in French). These Malga cheeses are often produced in small traditional dairies annexed to the temporary farms and are, therefore, very distinctive mountain cheeses [22,23,24].

Mountain cheeses ripened for only 3 weeks to 3 months (Asiago fresco, Montasio fresco, Monte Veronese latte intero, and Morlacco del Grappa) are produced from full-fat milk and often undergo a mild curd-cooking phase at 42–46 °C (Table 1 and Table 2). The size of the wheels is variable, but they are, on average, between 5 and 14 kg (Table 3 and Table 4). The mountain cheeses ripened over a long period (Asiago stagionato mezzano, vecchio and stravecchio; Montasio mezzano, and stagionato; Monte Veronese d’allevo mezzano and vecchio; Piave) are instead produced with partly skimmed milk, the curd is cooked at 44–48 °C (Table 1 and Table 2), and they are of medium size with little variation (5 to 9 kg; Table 3 and Table 4).

In terms of the length of ripening, the composition, acidity, and texture, mountain cheeses differ little from the cheeses produced in the plains (see “other cheeses”, Table 5, Table 6, Table 9 and Table 10). The most distinctive trait of mountain cheeses, and particularly those produced from pasture-grazed cows, is the color of the paste [25]. Among the 37 cheese categories compared (flavored cheeses excluded), Malga fresco has the yellowest paste, followed by Monte Veronese latte intero, and, among the long-ripened cheeses, Malga vecchio (Table 7 and Table 8).

### 4.3. Characteristics of Grana Padano Cheeses

For the most part, Grana Padano is manufactured in the plains from milk produced mainly on intensive farms that use total mixed rations, including corn silage and concentrates [12,26,27], but it is also produced in mountain areas in traditional farming systems [28,29]. Approximately one-quarter of all the milk produced in Italy goes to making Grana Padano [30], which is the PDO cheese with the highest level of production in Europe. Compared with long-ripened mountain cheeses, the ripening period is very long (Table 1), the milk is more thoroughly skimmed, the curd-cooking temperature is higher (56 °C), the salting period in brine is longer (Table 1), and the wheels are very heavy (32 to 40 kg each, Table 3). These distinctive cheese-making characteristics give the cheese paste not only its typical “grana” texture, but also a higher moisture content, a lower fat:protein ratio, and a higher WSN content (Table 5) compared with long-ripened mountain cheeses, although the color (Table 7), acidity, and firmness (Table 9) are similar. The chemical composition was similar to that found in previous research [26]

### 4.4. Characteristics of Pasta Filata Cheeses

Stretching the curd in very hot whey or water [12] is a technique used in the Veneto region and other Italian regions to make two PDO cheese categories (Provolone Valpadana dolce and piccante; Table 1), and three non-PDO categories (Mozzarella, Pasta filata mole, and Pasta filata; Table 2). There is a very wide variability in the shape, size, and ripening length in pasta filata cheeses. Fresh Mozzarella cheese alone comes in balls (ranging from 30 g for “bocconcini” to 125 g for regular balls and 500 g for large balls), braids/plaits (from 60 g for small “nodini” to 1 kg for large “treccia”), as well as blocks and cylinders (from 0.5 to 5 kg, used mainly in cooking pizza). However, pasta filata cheeses can also be produced for ripening (Provolone Valpadana, Table 1 and Pasta filata, Table 2). A typical Provolone is shaped like a large pear with a characteristic round knob for hanging it up and often weighs approximately 5–10 kg (Table 3 and Table 4), but it can also be cylindrical in shape and much heavier. The composition of pasta filata cheeses differs little from other cheese types, after taking length of ripening into account (Table 5 and Table 6), and only the soluble nitrogenous compound content seems to be smaller. As Mozzarella is very fresh and is eaten as it is, i.e., not intended for cooking or industrial use, the examples exhibited at *Caseus Veneti* had a higher moisture content than the cheeses reviewed by Ah and Tagalpallewar [31], as well as a higher ash content [4]. The color is very white and bright in fresh cheeses and more yellowish in ripened ones (Table 7 and Table 8).

### 4.5. Characteristics of Flavored Cheeses

The use of other substances in addition to milk, coagulant, and salt to modify the appearance, odor, and taste of cheese is widespread in northeast Italy. A traditional cheese recognized by the Italian Veneto region is the Formaggio inbriago (literally “drunken cheese”), which is matured in a covering of marc or immersed in must or red wine. Among the categories of cheeses without official recognition, the major flavored cheeses are the “Formaggi al pepe o peperoncino”, which are made by adding spices (black pepper grains or crushed chili pepper) to the curd before shaping. The second category, “Formaggi con erbe, fieno e spezie”, includes cheeses made with the addition of other spices and herbs to the curd before shaping or to the cheese surface after shaping (garlic, chives, olives, crushed nuts, etc.), as well as cheeses matured in hay, tree leaves, etc. The third category includes cheeses treated with different types and means of smoking (“Formaggi affumicati”), while the fourth category comprises blue cheeses (“Formaggi erborinati”), obtained from inoculation of molds, usually *Penicillium*. Lastly, a category was more recently created that included cheeses matured in beer or balsamic vinegar (“Formaggi alla birra, balsamico” similar to Formaggio inbriago).

These cheeses vary considerably from producer to producer and according to the area of production. They are generally made as wheels, but are also made as blocks. They are medium-small in size (often in the range of 2–4 kg), but larger cheeses are also found, especially among the categories of Formaggio inbriago and Formaggi alla birra e balsamico. Their composition reflects the fact that they are generally made from full-fat milk, with a highly variable length of ripening. Their appearance is obviously affected by the type, quantity, and distribution of the spices and herbs and the ripening conditions. The Formaggio inbriago has a very distinctive appearance, often with its dark violet rind and sometimes also its paste. The Formaggi erborinati (blue cheeses) are distinctive not only for the appearance of their rind and paste, which have a high yellowness index, but also for their chemical composition. They had the highest levels of WSN (Table 6) and pH of all the 37 cheese categories, confirming a well-known characteristic of this type of cheeses related to the metabolic activities of molds during ripening [17]. This peculiarity is also reflected by the inability of common “cheese” infrared calibrations to reliably determine the chemical composition of blue cheeses [13].

### 4.6. Characteristics of the Goat Cheeses

Goats’ milk stands out from the other dairy species, not only for its composition, but also for the coagulation and, especially, the curd-firming and syneresis processes [32,33,34,35]. In fact, goats produce a milk which coagulates faster than cows’ milk, and similarly to buffalo and sheep’s milk, but curd-firming is slower, the maximum curd firmness is lower, but syneresis is faster than the other ruminant species. Moreover, the goats reared in northern Italy are Alpine breeds (Saanen, Camosciata delle Alpi) and crossbreds, which differ from Mediterranean breeds in that their milk has a lower total solids content, delayed coagulation, and weaker curd [33,36,37]. This explains why, alongside bovine-like, rennet-coagulation cheese-making techniques, acid coagulation of goats’ milk is also used in Alpine areas, but not often in the Mediterranean region. Rennet coagulation of goat’s milk (“Caprino a coagulazione presamica” category) produces cheeses that apparently differ little from bovine cheeses produced with similar procedures, in terms of shape, size, composition, and firmness (Table 4, Table 6 and Table 10), but have a whiter paste (lower b* and C* and higher h° indices; Table 8).

Acid coagulation (“Caprino a coagulazione acida” category, Table 2) yields very different cheeses that are digested differently by humans [38]. They are sold very fresh, are very small (Table 4), and have variable shapes (small wheels, cylinders, blocks, etc.). These are the whitest of cheeses, with the highest L* and h° and the lowest b* and C* values (Table 8), and the texture is so soft that it is often not possible to measure it, and the cheese can also be spread (Table 10). Of course, the chemical composition of acid-coagulated goat cheeses is also very particular, not only for their very low pH (Table 10) [17] but also for having the highest moisture content, and, correspondingly, the lowest contents of fat, protein, WSN (except for Mozzarella), and ash (Table 6). It is worth noting that the property of acid coagulation to retain a greater quantity of water in the curd depends on the species of the milk. Comparing acid and rennet coagulation in the same laboratory conditions, we found evidently greater water retention with bovine milk but not bubaline milk [39].

### 4.7. Implications of the Study and the Need for Future Research

As part of the *Caseus Veneti* project, we were able to sample and compare more than 1000 cheeses, differing in type, producer, and year of production. This allowed us to gain insights into the enormous variability in the production and quality of cheese in real-world conditions, and to create a database for further research related to the effects of different cheese-making techniques on the quality and value of cheeses. The analysis of the major sources of variation allowed us to see that the first distinction among PDO cheeses (regulated by official norms of production approved by the European Union) [10], traditional cheeses, and other cheeses affects especially cheese moisture, protein, lightness, firmness, and adhesiveness. This is especially due to the fact that “other cheeses” groups the cheese categories not officially regulated, and particularly the cheeses freely designed by producers [9] to follow the changes in consumer requirements toward more “fresh” cheeses and new tastes obtained with the use of spices, nuts, and other ingredients [40]. The group of cheese categories are important sources of variation first of all for cheese weight, fat content, and color traits (lightness excluded). This is particularly true for groups having a PDO or traditional designation because of the norms regulating their production across all producers [10]. In any case, the differences among single cheese categories represent the major source of variation for all cheese traits, with the only exception of wheel weight and the maximum importance for ash content and cheese pH.

The interrelated nature of many of the cheese traits considered calls for a multivariate approach aimed at studying the correlations among different traits, identifying the latent explanatory factors underlying the complex relationships among cheese characteristics, and clustering and discriminating different types of cheeses.

The complexity of the traits describing the quality of the many types of cheese highlighted in this study also makes clear the need for more rapid and less labor-intensive secondary prediction methods than the usual wet laboratory methods. The results for chemical composition and color traits are promising, but not for textural properties; however, they also make clear the importance of analyzing a large variety of cheeses in order to identify the limits and biases of the secondary methods, which are often correlative in nature [14].

The traits considered in this study provide a wide but rough picture of cheese characteristics. To broaden our knowledge of cheese quality, more detailed studies are needed in several directions. The first is the analysis of human-health-related traits, and particularly the detailed profile of cheese fatty acids, particularly now that evidence is emerging of the scant value for human health of large categories of fatty acids, such as saturated-monounsaturated-polyunsaturated, omega3-omega6 and their ratio, trans-fatty acids, etc., and the need to quantify individual fatty acids, even those present in small amounts [41,42,43].

Detailed fatty acid profiles of cheeses could also be useful for estimating their environmental footprint, and particularly for predicting the enteric methane emissions (EME) of cows producing milk for cheese making, and we found that the equations derived from milk analyses [44] could also be used with satisfactory results on the fatty acid profiles of other dairy products, cheese included, especially if fresh or matured for up to 6 months [45,46].

Another very important research area is the sensory evaluation of cheese and its relationship with type of milk, cheese-making procedure, ripening length and conditions, etc. Analysis of volatile organic compounds (VOCs) is an important instrument for characterizing the flavor of cheese [47], sheds light on how their composition changes during the cheese-making and ripening processes, and could also be a useful tool for certifying labeled cheeses (geographic designations, organic agriculture, etc.) and, therefore, promoting them [48].

## Figures and Tables

**Table 1:** Main characteristics and cheese-making techniques of 16 cheese categories under the 7 protected designation of origin denominations (PDO, 321 cheeses) according to their production regulations.

PDO CHEESE Cheese Category	N	Fat ^1^	Heat ^2^	Culture Type ^3^	Curd Cutting Size ^4^	Curd Cooking °C	Curd Pressing	Cheese Salting ^5^	Ripening Time (Months)	Paste Firmness ^6^	Eyes
ASIAGO:	115										
-fresco (pressato)	45	full-fat	yes/no	milk	walnut	44 ± 2	yes	var	<1	soft	large
-stagionato-mezzano	28	skim	yes/no	milk	hazel	47 ± 2	yes	brine	4–10	firm	small
-stagionato-vecchio	19	skim	yes/no	milk	hazel	47 ± 2	yes	brine	10–15	hard	rare
-stagionato-stravecchio	23	skim	yes/no	milk	hazel	47± 2	yes	brine	>15	v-hard	rare
CASATELLA TREV. ^7^	27	full-fat	yes	milk	walnut	no	yes	var	0.2	v-soft	rare
GRANA PADANO:	35										
-Grana Padano	17	skim	no	whey	rice	56	no	brine	12–20	v-hard	no
-Grana Padano riserva	18	skim	no	whey	rice	56	no	brine	>20	v-hard	no
MONTASIO:	72										
-fresco	29	full-fat	yes/no	milk, st	rice	42–48	yes	salt/brine	2–4	soft	small
-mezzano	23	full-fat	yes/no	milk, st	rice	42–48	yes	salt/brine	5–10	firm	small
-stagionato	20	full-fat	yes/no	milk, st	rice	42–48	yes	salt/brine	>10	hard	small
MONTE VERONESE:	52										
-latte intero	21	full-fat	yes/no	milk, st	rice	43–45	no	brine	0.8–1.5	soft	med.
-d’allevo-mezzano	18	skim	no	milk, st	rice	46–48	no	brine	3–6	firm	small
-d’allevo-vecchio	13	skim	no	milk, st	rice	46–48	no	brine	>12	hard	small
PIAVE	6	3.5%	yes	milk/whey	rice	44–47	yes	brine	1–24	s-hard	no
PROVOLONE ^8^:	14										
-dolce	7	full-fat	yes	whey, st	var	-	no	brine	2–3	soft	small
-piccante	7	full-fat	yes	whey, st	var	-	no	brine	≥3	firm	small

^1^ Milk fat content: full-fat = milk not skimmed; skim = partly skimmed milk; var = varies according to dairy. ^2^ Heat treatment of milk: yes/no = varies according to dairy. ^3^ Microbiological culture used: st = industrial starter culture; milk = natural milk cultures; whey = natural whey cultures. ^4^ Size of curd particles after curd cutting: walnut = walnut; hazel = hazelnut; rice = rice grain; var = varies according to dairy. ^5^ Method of salting used: vat = salt added to milk in the vat; salt = dry salt on cheese surface; brine = immersion in brine; var = varies according to dairy. ^6^ Firmness of cheese paste: s-soft = semi soft; v-soft = very soft; s-hard = semi-hard; v-hard = very hard. ^7^ Casatella Trevigiana, a rindless cheese. ^8^ Provolone Valpadana, pasta filata (stretched curd cheese).

**Table 2:** Main characteristics and cheese-making techniques of the 4 traditional, 3 pasta filata, 5 flavored, 2 goat, and 7 other cheese categories without PDO status (*n* = 729 cheeses).

CHEESE GROUP Cheese Category	N	Milk Fat ^1^	Heat Treat ^2^	Cheese Ripening Time (Months)	Cheese Paste Firmness	Notes
TRADITIONAL CHEESES:	136					
-Morlacco del Grappa	30	skim	yes/no	1–3	soft	from Monte Grappa
-Malga fresco	42	skim	no	2–6	firm	from Alpine pastures
-Malga vecchio	19	skim	no	>12	very hard	from Alpine pastures
-Formaggio inbriago	45	full-fat	yes/no	2–6	firm	in marc/must/wine
PASTA FILATA CHEESES:	44					
-Mozzarella	20	full-fat	yes	<1	fresh	stretched curd cheese
-Pasta filata molle	13	full-fat	yes	1–3	soft	stretched curd cheese
-Pasta filata	11	full-fat	yes/no	3–12	hard	stretched curd cheese
FLAVORED CHEESES:	128					
-Formaggi al pepe o peperoncino	40	full-fat	yes	var.	var.	with pepper or chili
-Formaggi con erbe fieno e spezie	43	full-fat	yes	var.	var.	with herbs, hay, or spices
-Formaggi affumicati	22	full-fat	yes	var.	var.	smoked cheeses
-Formaggi erborinati	19	full-fat	yes	var.	var.	blue cheeses
-Formaggi alla birra o balsamico	4	full-fat	yes	var.	var.	matured in beer or balsamic vinegar
GOAT CHEESES:	54					
-Caprino a coagulazione acida	19	full-fat	yes	<1	fresh	acid coagulation
-Caprino a coagulazione presamica	35	full-fat	yes	var.	var.	rennet coagulation
OTHER CHEESES:	367					
-Freschi, freschissimi	57	full-fat	yes	<1	fresh	very fresh, no rind
-Caciotta	55	full-fat	yes	1–2	soft	<1.0 kg
-Latteria	79	full-fat	yes	2–4	semi-soft	>1.0 kg
-Crosta fiorita	25	full-fat	yes	1–4	soft	moldy rind
-Crosta lavata	18	full-fat	yes	2–6	semi-soft	washed rind
-Pasta semidura	91	var	yes/no	3–6	firm	-
-Pasta dura	42	var	yes/no	>10	hard	-

^1^ Milk fat content: full-fat = milk not skimmed; skim = partly skimmed milk; var = varies according to dairy. ^2^ Heat treatment of milk: yes/no = varies according to dairy. Cheese paste firmness: var = varies according to dairy.

**Table 3:** Shapes and sizes of the cheeses with EU Protected Designation of Origin (PDO) status (averages and standard deviations by cheese category).

PDO CHEESE Cheese Category	Shape	Height cm	Diameter cm	Weight kg
ASIAGO:				
-fresco (pressato)	wheel	14.5 ± 0.9	36.2 ± 1.7	13.9 ± 0.6
-stagionato-mezzano	wheel	10.0 ± 0.7	32.9 ± 0.9	9.1 ± 0.5
-stagionato-vecchio	wheel	9.7 ± 0.3	32.6 ± 1.1	8.8 ± 0.3
-stagionato-stravecchio	wheel	9.1 ± 0.6	31.8 ± 0.7	7.9 ± 0.6
CASATELLA TREV.	wheel	5.3 ± 1.0	13.6 ^1^ ± 6.6	1.1 ± 1.0
GRANA PADANO:				
-Grana Padano	wheel	22.5 ± 0.5	42.3 ± 3.4	34.7 ± 3.2
-Grana Padano riserva	wheel	22.1 ± 0.6	40.4 ± 0.9	34.6 ± 3.9
MONTASIO:				
-fresco	wheel	7.9 ± 0.6	29.9 ± 0.9	6.0 ± 0.5
-mezzano	wheel	7.7 ± 0.5	29.9 ± 1.1	5.8 ± 0.5
-stagionato	wheel	7.4 ± 0.5	29.6 ± 1.5	5.5 ± 0.5
MONTE VERONESE:				
-latte intero	wheel	9.3 ± 0.4	33.5 ± 1.3	8.6 ± 0.9
-d’allevo-mezzano	wheel	9.5 ± 0.5	31.3 ± 1.2	8.0 ± 1.0
-d’allevo-vecchio	wheel	9.0 ± 0.3	31.3 ± 0.8	7.6 ± 0.2
PIAVE	wheel	7.3 ± 0.4	29.0 ± 0.8	5.5 ± 0.1
PROVOLONE:				
-dolce	pear	11.8 ± 0.4	21.4 ^1^ ± 1.5	5.4 ± 0.2
-piccante	pear	19.5 ± 2.1	22.1 ^1^ ± 1.9	9.5 ± 0.3

^1^ length.

**Table 4:** Shapes and sizes of the non-PDO cheeses (averages and standard deviations by cheese category).

CHEESE GROUP Cheese Category	Shape	Height cm	Diameter cm	Weight kg
TRADITIONAL CHEESES:				
-Morlacco del Grappa	wheel	7.7 ± 1.1	29.7 ± 3.2	5.1 ± 0.8
-Malga fresco	wheel	8.4 ± 1.7	26.9 ± 5.8	5.3 ± 3.4
-Malga vecchio	wheel	8.8 ± 1.1	29.1 ± 5.9	6.4 ± 2.3
-Formaggio inbriago	variable	8.1 ± 0.8	27.8 ± 4.0 ^1^	5.4 ± 1.4
PASTA FILATA CHEESES:				
-Mozzarella	variable	-	-	0.2 ± 0.2
-Pasta filata molle	variable	-	-	0.3 ± 0.2
-Pasta filata	variable	-	-	5.0 ± 5.1
FLAVORED CHEESES:				
-Formaggi pepe, peperoncino	wheel	8.0 ± 2.1	17.2 ± 6.2	2.4 ± 1.8
-Formaggi con erbe fieno spezie	wheel	6.8 ± 2.1	16.2 ± 6.5	1.9 ± 1.8
-Formaggi affumicati	wheel	7.2 ± 1.1	21.4 ± 9.8	2.8 ± 2.6
-Formaggi erborinati	wheel	10.6 ± 2.5	16.0 ± 5.9	1.9 ± 1.1
-Formaggi alla birra, balsamico	wheel	8.5 ± 1.0	27.0 ± 6.4	5.1 ± 1.8
GOAT CHEESES:				
-Caprino coagulazione acida	variable	3.8 ± 0.6	7.0 ± 2.8 ^1^	0.2 ± 0.1
-Caprino coagulazione presamica	wheel	9.8 ± 4.7	20.8 ± 7.5	4.6 ± 1.4
OTHER CHEESES:				
-Freschi, freschissimi	variable	5.5 ± 2.2	12.2 ± 5.3 ^1^	0.8 ± 0.7
-Caciotta	wheel	6.8 ± 1.1	14.4 ± 4.1	1.1 ± 0.8
-Latteria	wheel	9.2 ± 3.1	30.0 ± 4.5	7.0 ± 3.4
-Crosta fiorita	wheel	5.2 ± 1.3	15.1 ± 7.9	1.4 ± 1.8
-Crosta lavata	wheel	7.7 ± 1.2	19.0 ± 9.8	2.3 ± 1.8
-Pasta semidura	wheel	8.8 ± 1.4	27.6 ± 6.0	5.8 ± 2.7
-Pasta dura	wheel	10.8 ± 4.7	31.1 ± 7.3	8.7 ± 2.3

^1^ length.

**Table 5:** Chemical compositions of the cheeses with EU Protected Designation of Origin (PDO) status (averages and standard deviations by cheese category, expressed as % by mass).

PDO CHEESE Cheese Category	Moisture %	Fat %	Protein %	WSN ^1^ %	Ash %
ASIAGO:					
-fresco (pressato)	41.0 ± 1.7	29.9 ± 1.6	23.5 ± 1.1	5.3 ± 0.4	1.9 ± 0.1
-stagionato-mezzano	34.4 ± 0.9	31.9 ± 1.1	29.7 ± 1.0	6.5 ± 0.2	2.0 ± 0.1
-stagionato-vecchio	31.5 ± 1.2	32.9 ± 1.6	31.4 ± 1.8	7.1 ± 0.3	2.0 ± 0.1
-stagionato-stravecchio	25.9 ± 2.3	35.5 ± 2.1	31.2 ± 2.2	8.4 ± 0.9	2.1 ± 0.1
CASATELLA TREV.	57.3 ± 1.5	21.9 ± 1.6	14.8 ± 0.9	3.3 ± 0.3	1.4 ± 0.2
GRANA PADANO:					
-Grana Padano	33.9 ± 1.5	27.8 ± 1.1	35.4 ± 1.3	9.8 ± 0.6	2.0 ± 0.1
-Grana Padano riserva	31.6 ± 1.3	28.4 ± 0.9	36.7 ± 0.6	10.4 ± 0.7	2.1 ± 0.0
MONTASIO:					
-fresco	35.4 ± 2.1	32.6 ± 1.5	27.1 ± 1.7	6.3 ± 0.5	2.0 ± 0.1
-mezzano	33.1 ± 1.0	34.2 ± 2.3	28.8 ± 1.7	6.9 ± 0.6	2.0 ± 0.1
-stagionato	28.2 ± 3.4	36.5 ± 2.3	28.1 ± 3.3	7.9 ± 0.7	2.0 ± 0.0
MONTE VERONESE:					
-latte intero	38.6 ± 2.3	29.7 ± 1.4	25.9 ± 1.1	5.6 ± 0.4	1.8 ± 0.1
-d’allevo-mezzano	33.8 ± 1.6	33.6 ± 1.0	27.8 ± 1.5	6.8 ± 0.5	2.0 ± 0.1
-d’allevo-vecchio	28.4 ± 2.4	34.6 ± 3.7	31.3 ± 3.9	7.4 ± 0.1	2.0 ± 0.0
PIAVE	27.8 ± 1.4	37.2 ± 0.1	29.2 ± 0.9	9.1 ± 1.3	2.1 ± 0.0
PROVOLONE:					
-dolce	40.8 ± 2.4	28.6 ± 3.3	25.0 ± 1.6	6.0 ± 0.1	2.0 ± 0.1
-piccante	37.2 ± 0.4	30.9 ± 1.0	25.3 ± 1.2	6.8 ± 0.8	2.1 ± 0.1

^1^: WSN = water-soluble nitrogen, expressed as % of total nitrogen.

**Table 6:** Chemical compositions of the non-PDO cheeses (averages and standard deviations by cheese category expressed as % by mass).

CHEESE GROUP Cheese Category	Moisture %	Fat %	Protein %	WSN ^1^ %	Ash %
TRADITIONAL CHEESES:					
-Morlacco del Grappa	50.9 ± 3.1	23.1 ± 2.7	19.4 ± 2.3	4.4 ± 0.5	2.0 ± 0.2
-Malga fresco	38.6 ± 4.4	29.0 ± 2.9	26.9 ± 2.3	6.3 ± 0.8	1.8 ± 0.1
-Malga vecchio	30.9 ± 3.3	30.7 ± 3.3	32.9 ± 3.7	7.6 ± 0.8	1.9 ± 0.1
- Formaggio inbriago	33.5 ± 2.9	33.1 ± 2.0	29.0 ± 3.0	7.8 ± 1.1	2.0 ± 0.1
PASTA FILATA CHEESES:					
-Mozzarella	60.8 ± 0.2	19.9 ± 2.4	15.9 ± 1.9	3.0 ± 0.3	1.3 ± 0.3
-Pasta filata molle	47.3 ± 13.2	27.9 ± 6.9	20.3 ± 5.0	4.2 ± 1.6	1.5 ± 0.4
-Pasta filata	38.3 ± 6.0	27.6 ± 4.2	27.6 ± 1.0	6.2 ± 1.5	2.0 ± 0.1
FLAVORED CHEESES:					
-Formaggi pepe, peperoncino	37.4 ± 4.7	31.0 ± 2.3	25.1 ± 3.5	7.9 ± 2.1	1.9 ± 0.2
-Formaggi con erbe fieno spezie	42.2 ± 16.0	28.2 ± 6.9	22.5 ± 8.0	6.6 ± 1.5	1.8 ± 0.3
-Formaggi affumicati	37.4 ± 4.9	30.5 ± 3.8	25.2 ± 1.7	6.0 ± 0.8	1.9 ± 0.1
-Formaggi erborinati	42.4 ± 5.0	25.7 ± 4.0	25.2 ± 3.9	9.4 ± 2.8	1.4 ± 0.5
-Formaggi alla birra, balsamico	35.5 ± 2.5	33.0 ± 1.8	26.8 ± 2.0	8.6 ± 2.8	2.1 ± 0.0
GOAT CHEESES:					
-Caprino coagulazione acida	66.3 ± 3.9	15.5 ± 2.8	12.9 ± 1.3	3.2 ± 0.3	1.2 ± 0.3
-Caprino coagulazione presamica	44.5 ± 9.4	25.2 ± 5.9	24.9 ± 5.6	6.3 ± 2.2	2.0 ± 0.3
OTHER CHEESES:					
-Freschi, freschissimi	56.4 ± 4.8	23.4 ± 4.0	14.5 ± 2.5	3.4 ± 0.4	1.5 ± 0.3
-Caciotta	43.3 ± 4.3	28.6 ± 3.4	21.9 ± 1.6	4.9 ± 0.7	1.9 ± 0.2
-Latteria	41.8 ± 3.4	29.9 ± 2.9	22.8 ± 2.4	5.3 ± 0.7	1.9 ± 0.2
-Crosta fiorita	48.9 ± 3.0	26.7 ± 3.0	19.2 ± 1.5	4.4 ± 0.6	2.0 ± 0.2
-Crosta lavata	41.2 ± 5.7	30.8 ± 6.2	22.0 ± 3.0	5.2 ± 1.4	2.0 ± 0.1
-Pasta semidura	35.5 ± 2.9	33.4 ± 5.9	25.9 ± 2.6	6.4 ± 0.9	2.0 ± 0.1
-Pasta dura	30.5 ± 3.2	34.1 ± 4.1	30.2 ± 3.6	8.5 ± 1.5	2.0 ± 0.1

^1^: WSN = water-soluble nitrogen, expressed as % of total nitrogen.

**Table 7:** Color traits ^1^ of the paste of the cheeses with EU Protected Designation of Origin (PDO) status (averages and standard deviations by cheese category).

PDO CHEESE Cheese Category	L*	a*	b*	C*	h°
ASIAGO:					
-fresco (pressato)	79.1 ± 2.0	−0.9 ± 0.5	13.7 ± 1.5	13.7 ± 1.5	93.9 ± 2.3
-stagionato-mezzano	71.8 ± 2.4	−1.1 ± 0.4	11.1 ± 1.4	11.2 ± 1.4	95.9 ± 2.6
-stagionato-vecchio	67.1 ± 4.0	−1.3 ± 0.2	11.9 ± 1.8	11.9 ± 1.7	96.4 ± 1.8
-stagionato-stravecchio	65.5 ± 4.1	−1.2 ± 0.5	10.4 ± 1.0	10.5 ± 1.0	96.5 ± 2.6
CASATELLA TREV.	84.0 ± 8.0	−1.5 ± 1.0	10.1 ± 1.9	10.3 ± 1.6	99.8 ± 9.7
GRANA PADANO:					
-Grana Padano	69.6 ± 4.4	−0.6 ± 0.4	11.3 ± 0.6	11.3 ± 0.6	93.2 ± 2.2
-Grana Padano riserva	67.5 ± 3.7	−0.6 ± 0.3	11.4 ± 1.3	11.4 ± 1.2	93.3 ± 1.8
MONTASIO:					
-fresco	76.7 ± 1.3	−0.9 ± 0.4	12.9 ± 1.6	13.0 ± 1.6	94.2 ± 2.2
-mezzano	73.4 ± 1.9	−0.9 ± 0.5	12.6 ± 2.3	12.6 ± 2.3	94.5 ± 3.1
-stagionato	68.3 ± 4.4	−1.5 ± 0.6	11.7 ± 2.0	11.8 ± 2.0	97.9 ± 3.9
MONTE VERONESE:					
-latte intero	74.1 ± 2.2	0.1 ± 1.1	17.5 ± 4.2	17.6 ± 4.1	90.6 ± 4.3
-d’allevo-mezzano	71.7 ± 2.2	−0.9 ± 0.6	11.3 ± 3.2	11.4 ± 3.1	95.3 ± 3.4
-d’allevo-vecchio	68.0 ± 3.4	−1.2 ± 0.8	10.1 ± 3.3	10.2 ± 3.2	98.2 ± 6.8
PIAVE	66.1 ± 1.7	−1.6 ± 0.2	8.6 ± 0.5	8.7 ± 0.5	100 ± 1.7
PROVOLONE:					
-dolce	79.4 ± 3.1	−1.0 ± 0.2	12.8 ± 0.1	12.8 ± 0.1	94.4 ± 0.9
-piccante	76.4 ± 1.7	−1.1 ± 0.6	11.7 ± 0.6	11.8 ± 0.5	95.2 ± 3.4

^1^: L* value is the lightness coefficient (ranging from black = 0 to white = 100); a* value indicates the position on the green (−) to red (+) axis; b* value indicates the position on the blue (−) to yellow (+) axis; C* (chroma) is calculated as $C{\;}^{*}=\sqrt{(a^{2}+b^{2}})$; h° (hue angle) values are calculated from the arctangent of b*/a*.

**Table 8:** Color traits ^1^ of the paste of the non-PDO cheeses (averages and standard deviations by cheese category).

CHEESE GROUP Cheese Category	L*	a*	b*	C*	h°
TRADITIONAL CHEESES:					
-Morlacco del Grappa	82.9 ± 3.0	−0.4 ± 0.6	13.8 ± 2.8	13.8 ± 2.8	92.0 ± 2.6
-Malga fresco	73.9 ± 5.2	0.3 ± 0.6	18.3 ± 2.7	18.4 ± 2.7	89.2 ± 1.9
-Malga vecchio	65.3 ± 4.0	−0.9 ± 0.7	14.4 ± 2.6	14.4 ± 2.5	93.8 ± 2.9
- Formaggio inbriago	72.0 ± 5.3	−1.2 ± 0.9	13.3 ± 2.7	13.4 ± 2.6	95.9 ± 5.3
PASTA FILATA CHEESES:					
-Mozzarella	89.3 ± 3.6	−1.4 ± 0.5	9.4 ± 1.4	9.5 ± 1.4	98.1 ± 2.5
-Pasta filata molle	82.7 ± 5.9	−1.9 ± 1.6	12.6 ± 2.8	12.8 ± 3.0	97.8 ± 4.3
-Pasta filata	78.8 ± 5.3	−1.8 ± 0.8	12.7 ± 1.1	12.9 ± 1.0	98.1 ± 4.2
FLAVORED CHEESES:					
-Formaggi pepe, peperoncino	76.9 ± 4.5	−0.1 ± 1.5	14.3 ± 3.2	14.4 ± 3.1	91.8 ± 7.7
-Formaggi con erbe fieno spezie	78.4 ± 7.9	−1.3 ± 0.9	12.8 ± 2.7	12.9 ± 2.6	96.7 ± 5.4
-Formaggi affumicati	77.8 ± 2.9	−0.9 ± 0.7	14.7 ± 2.4	14.8 ± 2.3	94.3 ± 3.9
-Formaggi erborinati	85.2 ± 9.9	−1.0 ± 1.6	16.1 ± 13.6	16.3 ± 13.4	98.1 ± 8.6
-Formaggi alla birra, balsamico	78.2 ± 7.7	−0.1 ± 1.7	14.3 ± 4.3	14.3 ± 4.4	91.4 ± 5.6
GOAT CHEESES:					
-Caprino coagulazione acida	91.9 ± 5.1	−1.6 ± 0.5	8.7 ± 1.5	8.9 ± 1.4	100 ± 4.1
-Caprino coagulazione presamica	80.9 ± 5.8	−1.8 ± 0.6	9.8 ± 1.8	10.0 ± 1.7	100 ± 4.5
OTHER CHEESES:					
-Freschi, freschissimi	88.8 ± 4.3	−1.0 ± 0.8	11.0 ± 2.4	11.1 ± 2.4	95.9 ± 4.4
-Caciotta	82.2 ± 4.1	−0.6 ± 1.1	14.7 ± 3.1	14.8 ± 3.1	92.7 ± 3.8
-Latteria	79.5 ± 4.2	−0.9 ± 0.8	14.1 ± 3.0	14.2 ± 3.0	93.9 ± 3.1
-Crosta fiorita	85.2 ± 2.8	−1.2 ± 0.8	12.3 ± 2.1	12.4 ± 2.0	95.9 ± 4.8
-Crosta lavata	80.9 ± 4.6	−1.3 ± 1.0	13.5 ± 2.4	13.6 ± 2.3	95.9 ± 4.6
-Pasta semidura	75.6 ± 3.5	−1.0 ± 0.4	13.5 ± 1.8	13.5 ± 1.8	94.3 ± 2.2
-Pasta dura	70.2 ± 7.4	−1.5 ± 0.8	10.9 ± 2.1	11.1 ± 2.0	98.3 ± 4.8

^1^: L* value is the lightness coefficient (ranging from black = 0 to white = 100); a* value indicates the position on the green (−) to red (+) axis; b* value indicates the position on the blue (−) to yellow (+) axis; C* (chroma) is calculated as $C{\;}^{*}=\sqrt{(a^{2}+b^{2}})$; h° (hue angle) values are calculated from the arctangent of b*/a*.

**Table 9:** Acidity and textural traits of cheeses with EU Protected Designation of Origin (PDO) status (averages and standard deviations by cheese category).

PDO CHEESE Cheese Category	pH	Firmness, N	Adhesiveness, J^−3^
ASIAGO:			
-fresco (pressato)	5.67 ± 0.19	5.5 ± 2.0	5.1 ± 2.8
-stagionato-mezzano	5.80 ± 0.19	15.9 ± 4.8	13.8± 4.9
-stagionato-vecchio	5.93 ± 0.32	19.0 ± 4.8	14.3 ± 5.7
-stagionato-stravecchio	5.96 ± 0.18	20.9 ± 5.1	13.4 ± 7.2
CASATELLA TREV.	5.56 ± 0.15	7.1 ± 6.4	5.8 ± 0.9
GRANA PADANO:			
-Grana Padano	5.95 ± 0.28	22.2 ± 5.9	10.2 ± 2.1
-Grana Padano riserva	5.98 ± 0.14	21.7 ± 12.6	10.1 ± 2.9
MONTASIO:			
-fresco	5.76 ± 0.15	12.1 ± 4.2	10.2 ± 2.8
-mezzano	5.87 ± 0.23	13.2 ± 3.9	10.3 ± 2.9
-stagionato	5.96 ± 0.31	17.6 ± 1.8	11.0 ± 1.0
MONTE VERONESE:			
-latte intero	5.78 ± 0.16	7.9 ± 2.9	7.1 ± 3.8
-d’allevo-mezzano	5.66 ± 0.30	10.8 ± 3.8	8.9 ± 2.6
-d’allevo-vecchio	6.06 ± 0.23	16.2 ± 7.6	9.2 ± 2.3
PIAVE	5.59 ± 0.13	26.0 ± 1.9	17.0 ± 1.2
PROVOLONE:			
-dolce	5.21 ± 0.07	13.5 ± 9.5	8.0 ± 2.1
-piccante	5.64 ± 0.03	11.3 ± 6.4	8.2 ± 7.5

**Table 10:** Acidity and textural traits of the non-PDO cheese categories (averages and standard deviations by cheese category).

CHEESE GROUP Cheese Category	pH	Firmness, N	Adhesiveness, J^−3^
TRADITIONAL CHEESES:			
-Morlacco del Grappa	5.36 ± 0.16	3.5 ± 3.1	3.2 ± 2.5
-Malga fresco	5.59 ± 0.19	6.4 ± 3.2	6.0 ± 2.8
-Malga vecchio	5.78 ± 0.15	14.9 ± 5.0	10.3 ± 4.5
- Formaggio inbriago	5.96 ± 0.18	12.5 ± 4.4	8.1 ± 2.9
PASTA FILATA CHEESES:			
-Mozzarella	5.63 ± 0.38	3.8 ± 5.8	2.1 ± 0.9
-Pasta filata molle	5.37 ± 0.27	-	-
-Pasta filata	5.75 ± 0.06	11.9 ± 3.7	10.2 ± 3.5
FLAVORED CHEESES:			
-Formaggi pepe, peperoncino	5.63 ± 0.16	8.7 ± 6.7	5.9 ± 4.4
-Formaggi con erbe fieno spezie	5.62 ± 0.33	8.7 ± 7.7	7.1 ± 8.3
-Formaggi affumicati	5.51 ± 0.14	4.9 ± 2.7	3.8 ± 2.2
-Formaggi erborinati	6.61 ± 0.23	6.7 ± 4.6	6.1 ± 3.2
-Formaggi alla birra, balsamico	5.41 ± 0.38	13.7 ± 8.9	7.9 ± 5.0
GOAT CHEESES:			
-Caprino coagulazione acida	4.70 ± 0.13	-	-
-Caprino coagulazione presamica	5.52 ± 0.20	7.9 ± 5.9	6.2 ± 4.8
OTHER CHEESES:			
-Freschi, freschissimi	5.57 ± 0.46	4.8 ± 4.7	3.8 ± 2.1
-Caciotta	5.44 ± 0.23	6.0 ± 5.0	6.2 ± 4.7
-Latteria	5.58 ± 0.13	5.6 ± 3.5	6.0 ± 3.3
-Crosta fiorita	5.77 ± 0.26	2.7 ± 2.3	3.3 ± 2.1
-Crosta lavata	5.51 ± 0.21	4.0 ± 3.0	3.1 ± 2.7
-Pasta semidura	5.54 ± 0.26	9.9 ± 4.4	8.3 ± 3.0
-Pasta dura	5.90 ± 0.25	18.5 ± 9.2	12.1 ± 7.4

## Data Availability

Data can be requested from authors.
